# Hyperacute Simultaneous Cardiocerebral Infarction: Rescuing the Brain or the Heart First?

**DOI:** 10.3389/fneur.2017.00664

**Published:** 2017-12-07

**Authors:** Naruchorn Kijpaisalratana, Aurauma Chutinet, Nijasri C. Suwanwela

**Affiliations:** ^1^Chulalongkorn Stroke Center, King Chulalongkorn Memorial Hospital, Bangkok, Thailand; ^2^Faculty of Medicine, Department of Medicine, Division of Neurology, Chulalongkorn University, Bangkok, Thailand

**Keywords:** cardiocerebral infarction, acute ischemic stroke, acute myocardial infarction, simultaneous, insular cortex

## Abstract

Concurrent acute ischemic stroke and acute myocardial infarction is an uncommon medical emergency condition. The challenge for the physicians regarding the management of this situation is paramount since early management of one condition will inevitably delay the other. We present two illustrative cases of “hyperacute simultaneous cardiocerebral infarction” who presented with simultaneous cardiocerebral infarction and arrived at the hospital within the thrombolytic therapeutic window for acute ischemic stroke of 4.5 h. We propose an algorithm for managing the patient with hyperacute simultaneous cardiocerebral infarction based on hemodynamic status and suggest close cardiac monitoring based on the site of cerebral infarction.

## Introduction

Both acute ischemic stroke and acute myocardial infarction are medical emergency conditions, which require timely diagnosis and management. It has been shown that acute ischemic stroke increases the risk of acute myocardial infarction and *vice versa* (1, 2). However, simultaneous acute ischemic stroke and acute myocardial infarction previously described as “cardiocerebral infarction” has been rarely reported (3). We propose the term “hyperacaute simultaneous cardiocerebral infarction” to describe patients with simultaneous cardiocerebral infarction who arrived at the hospital within 4.5 h of the thrombolytic therapeutic window. Due to the rarity of the condition, the management of these patients is very challenging and there is no ideal recommendation. Balanced management should be a trade-off between early rescuing the brain or the heart.

From our experience at King Chulalongkorn Memorial Hospital, we present two cases of hyperacute simultaneous cardiocerebral infarction and propose an algorithm for patient management in this challenging situation.

## Case Illustrations

### Case 1

**Figure 1:**
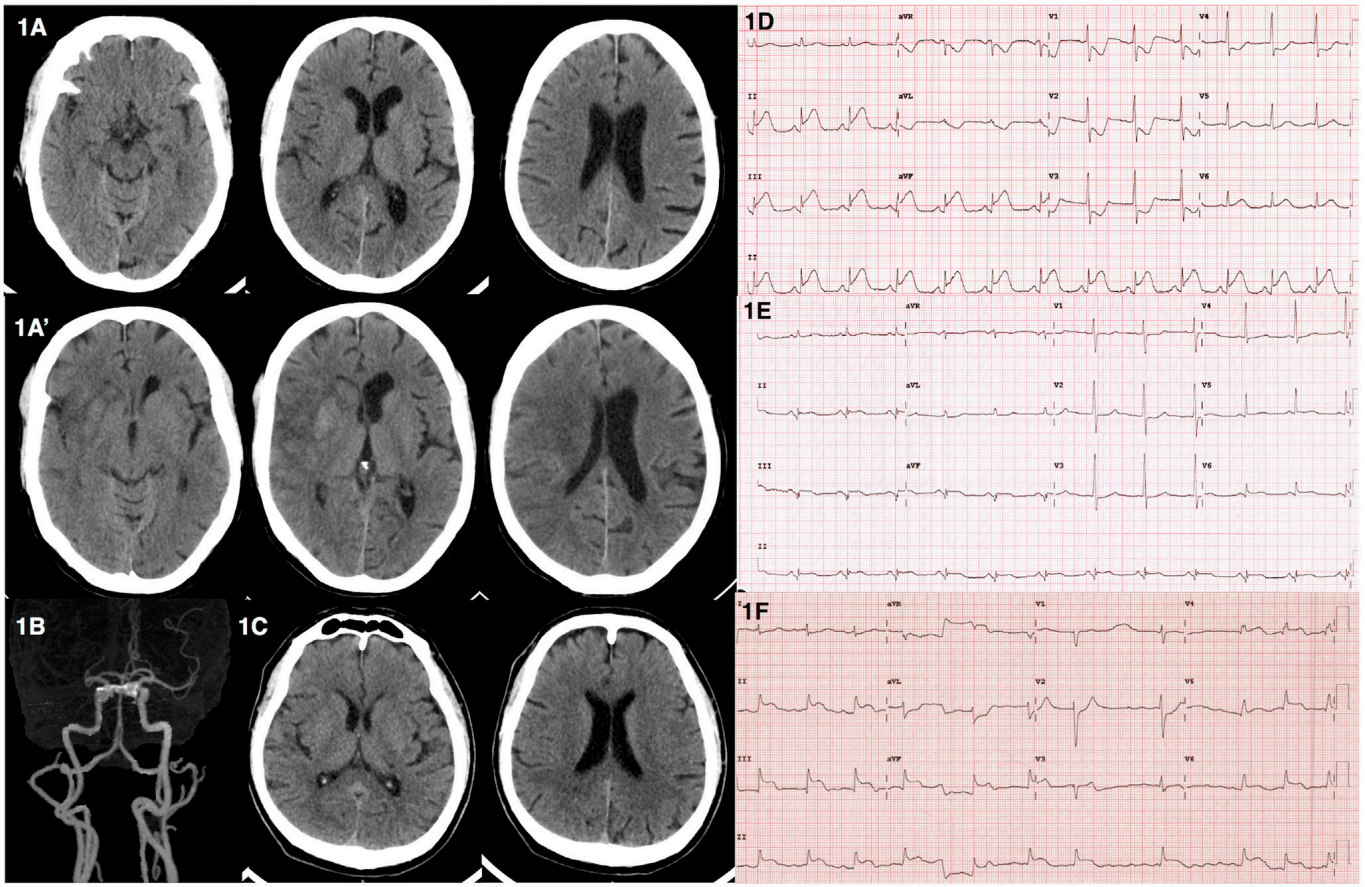
Computed tomography (CT) and electrocardiographic findings from illustrative cases. **(A)** Case 1**––**CT brain revealed subtle signs of acute right-middle cerebral artery infarction including loss of insular ribbon and basal ganglia hypoattennuation. **(A′)** Case 1**––**follow-up CT brain revealed interval increase hypodensity lesion with gyral effacement involving right insular cortex with hemorrhagic transformation in right caudate and putamen. **(B)** Case 1**––**CT angiogram showed occlusion of proximal to mid right M1 of middle cerebral artery. **(C)** Case 2**––**CT brain showed no acute ischemic or hemorrhagic lesions. **(D)** Case 1**––**electrocardiogram showed ST elevation in leads II, III, aVF. **(E)** Case 1**––**electrocardiogram showed resolution of ST elevation after PCI. **(F)** Case 2**––**electrocardiogram showed junctional rhythm with ST elevation in leads II, III, aVF, and V4–6.

A 65-year-old male presented to the emergency department due to acute onset of left hemiparesis, spastic dysarthria, gaze preference to the right and left hemineglect while waiting for radiotherapy for his underlying disease of right parotid gland carcinoma in the hospital. He was alert, oriented, and able to follow commands. He had a National Institute of Health Stroke Scale of 12. His hemodynamic status was stable with a blood pressure of 110/68 mmHg and pulse rate of 74 bpm. He did not complain of chest discomfort. Computed tomography (CT) of the brain revealed subtle signs of acute ischemic stroke in the right-middle cerebral artery territory (Figure 1A). CT angiogram showed occlusion at proximal to mid M1 segment of the right-middle cerebral artery (Figure 1B). Intravenous recombinant tissue plasminogen activator (rtPA) was administered with standard dose of 0.9 mg/kg at 2 h 35 min after onset. During the thrombolytic administration, an electrocardiogram was performed and showed ST elevation in leads II, III, and aVF (Figure 1D). Initial cardiac troponin I was 137.4 pg/mL (<34.2 pg/mL) and creatine kinase-MB (CKMB) was 27 U/L (0–24 U/L). Due to the active cardiac condition and large infarct core from the CT perfusion the neuro-interventionist decided not to perform mechanical thrombectomy after intravenous thrombolytic despite the large vessel occlusion. The emergency coronary angiography was performed and revealed total occlusion at mid left circumflex artery. Percutaneous coronary intervention (PCI) with drug eluting stent placement was performed. Thrombolysis in myocardial infarction (TIMI) grade flow of 3 (complete perfusion) was achieved after the cardiac intervention. The follow-up electrocardiogram showed resolution of the ST-segment elevation (Figure 1E). Serial cardiac enzyme following next 5 h revealed an elevation of both serum cardiac troponin I and CKMB of 4,643 pg/mL and 325 U/L, respectively. An echocardiogram revealed mild basal to mid inferoposterior wall hypokinesia with normal left ventricular ejection fraction of 62%. He was admitted to the coronary care unit for 1 day and then transferred to the stroke unit. The neurological deficits were unchanged. A follow-up CT scan at 24-h post-thrombolytic therapy revealed an ischemic stroke in the right-middle cerebral artery territory involving in right insular cortex, right lentiform nucleus, and right-frontal region (Figure 1A′).

### Case 2

A 64-year-old male presented with acute onset of left hemiparesis and concurrent chest pain 1.5 h prior to hospital arrival. His initial hemodynamic status was stable with a blood pressure of 107/84 mmHg and pulse rate of 84 bpm. He was alert and cooperative. He had a forced gaze deviation to the right, left hemiplegia, left hemianesthesia, and dysarthric speech. He had a National Institute of Health Stroke Scale of 13. A CT scan of the brain showed no evidence of hemorrhagic or acute ischemic lesions (Figure 1C). The neurological deficits were compatible with proximal right-middle cerebral artery occlusion. An electrocardiogram revealed junctional rhythm with ST elevation in leads II, III, aVF, and V4-V6 (Figure 1F). A bedside echocardiogram revealed severe global hypokinesia with an ejection fraction of 30%. The patient then developed sudden cardiac arrest prior to the administration of rtPA. The electrocardiogram showed pulseless electrical activity interchange with ventricular fibrillation during the cardiopulmonary resuscitation. He was transferred to the angiography suite for emergency PCI. Coronary angiogram revealed total occlusion of the middle part of the left anterior descending coronary artery and 99% stenosis of the proximal right coronary artery. Balloon angioplasty at the proximal right coronary artery was performed with unsuccessful recanalization. The residual stenosis was 80%. Despite the treatment, there was no returning of the spontaneous circulation after 70 m of cardiopulmonary resuscitation.

## Prevalence of Ischemic Stroke and Myocardial Infarction

It has been known that following a transient ischemic attack (TIA) and acute stroke, there is an increased risk of acute myocardial infarction. After TIA, average annual incidence of myocardial infarction was 1%, whereas the relative risk for myocardial infarction among patients with TIA was 2.09 (95% CI, 1.52–2.81) compared with the general population (4). According to the Austrian stroke unit registry, during treatment in the stroke unit (median duration 3 days), 1% of patients with TIA or ischemic stroke and 0.3% of patients with hemorrhagic stroke suffered from myocardial infarction (5). A prospective observational study by Mochmann et al. revealed that approximately 13.7% of patients with acute ischemic stroke had elevated level of cardiac troponin. However, compared with age-and gender-matched patients with non-ST-elevation acute coronary syndrome (NSTE-ACS), coronary culprit lesions were significantly less frequent among acute ischemic stroke patients (6).

On the other hand, several studies have demonstrated the prevalence as well as risk factors of acute ischemic stroke after acute myocardial infarction (2, 7, 8). Following an acute coronary syndrome, in-hospital stroke occurred approximately 0.9% with the highest incidence among patients with ST-segment elevation myocardial infarction (7). The risk of acute stroke is highest within 5 days after acute myocardial infarction (9).

“Cardiocerebral infarction” was first described in 2010 by Omar et al. in a case report of concomitant acute ischemic stroke and acute myocardial infarction (3). Although there is an increased risk of acute myocardial infarction following acute stroke and *vice versa*, simultaneous cardiocerebral infarction has rarely been reported, with an incidence of 0.009% (10). Cardiocerebral infarction can be classified as “synchronous cardiocerebral infarction” which is a simultaneous infarction in the cerebral and coronary vascular territories, and “metachronous cardiocerebral infarction” which is one event preceding the other (10). Metachronous cardiocerebral infarction is not the main focus in this article since the management is based on the sequence of the thrombotic event accordingly.

Simultaneous cardiocerebral infarction can be diagnosed by the presence of simultaneous acute onset of a focal neurological deficit, indicating acute stroke and a chest pain or evidence of myocardial infarction such as changes of electrocardiogram and the elevation of cardiac enzymes (3, 10–13). So far, eight cases of “hyperacute simultaneous cardiocerebral infarction” have been reported (3, 10–13). All of the ischemic stroke among patients with simultaneous cardiocerebral infarction occurred in large vessel territories. ST-segment elevation in inferior leads was the most common electrocardiographic findings among these patients. Managements and clinical outcomes were varied among cases.

## Pathophysiology of Simultaneous Cardiocerebral Infarction

Pathophysiology of simultaneous cardiocerebral infarction can be classified into three categories: (1) conditions leading to concurrent cerebral–coronary infarction, (2) cardiac conditions leading to cerebral infarction, and (3) brain–heart axis dysregulation or cerebral infarction leading to myocardial infarction.

There are several conditions that lead to simultaneous acute cerebral and coronary infarction. Atrial fibrillation has been reported as a cause of simultaneous cardiocerebral infarction due to common source of both cerebral and coronary embolism (14, 15). Type-I aortic dissection with dissection flap extending to coronary and common carotid arteries origin had been reported to cause concurrent acute myocardial infarction and acute ischemic stroke (16). In addition, concurrent coronary and cerebral vasospasm due to electrical injury have been reported as an uncommon cause of simultaneous cardiocerebral infarction (17).

Pre-existing intracardiac thrombus from poor left ventricular ejection fraction can also lead to simultaneous coronary and cerebral vascular occlusion (10). Likewise, a thrombus formed in the right ventricle in acute right ventricular infarction with right ventricular failure in combination with patent foramen ovale can embolize to both vascular territories (3). Severe hypotension following acute myocardial infarction can also lead to hemodynamic stroke (3, 10).

Brain–heart axis dysregulation might be an alternative pathophysiology of simultaneous cardiocerebral infarction. It has been shown that the insular cortex plays a critical role in central autonomic system regulation (18). Pathology in the insular cortex has been associated with arrhythmia, myocardial injury, and disruption of diurnal blood pressure variation (18). Patients with acute ischemic stroke in the parietoinsular region were found to have higher risk of developing atrial fibrillation (19). An abnormal electrocardiogram, including ST-segment elevation, was found to be related to ischemic stroke in the insular cortex (20). In addition to electrocardiographic abnormalities, myocardial injury determined by serum cardiac troponin T elevation was shown to be associated with cerebral infarction in specific brain regions including the right insular and right inferior parietal lobule (21). Cardiac sympathetic overactivity from an insular cortex lesion can provoke diffuse myocardial damage, “myocytolysis,” which leads to cardiac enzyme elevation (21, 22). Results from human studies showed that the stimulation of different sides of the insular cortex resulted in different cardiac autonomic responses. The right-side stimulation resulted in a predominant sympathetic effect, whereas the left-side stimulation resulted in a predominant parasympathetic effect (18, 21).

Interestingly, seven out of eight reported cases of hyperacute simultaneous cardiocerebral infarction had ischemic stroke involved in the insular cortex due to middle cerebral artery occlusion (10–13). Both of our cases also had ischemic stroke involved in the insular cortex due to middle cerebral artery occlusion.

Case 1 had concurrent right-middle cerebral artery and left circumflex coronary artery occlusion. We propose that the possible mechanism of simultaneous cardiocerebral infarction in this patient was primarily from acute ischemic stroke in the right-middle cerebral artery territory including the right insular region. The right insular infarction could enhance cardiac sympathetic activity and myocardial injury especially if the patient had pre-existing atherosclerotic coronary artery disease. Alternatively, right insular infarction might cause paroxysmal atrial fibrillation, which led to acute coronary embolism. Case 2 had simultaneous right-middle cerebral artery, right coronary artery, and left anterior descending coronary artery occlusion. The pre-existing intracardiac thrombus from low left ventricular ejection fraction might be the source of both cerebral and coronary emboli.

## Hyperacute Simultaneous Cardiocerebral Infarction: Management and Dilemma

Due to the rarity of simultaneous cardiocerebral infarction, recommendation for optimal reperfusion strategy in this scenario is still lacking. A case of hyperacute simultaneous cardiocerebral infarction presents challenges and dilemmas for the physician. The initial treatment of one condition will inevitably delay the other. Intravenous thrombolytic therapy with rtPA is a standard treatment for acute ischemic stroke for patients who arrived within 4.5 h after onset unless contraindicated (23). Primary PCI is the first-line reperfusion therapy for patients with acute ST-elevation myocardial infarction (STEMI) and selected patients with NSTE-ACS including those with refractory angina, hemodynamically, or electrically unstable (24, 25). Fibrinolytic therapy within 12 h after onset is an alternative treatment for STEMI in the setting of non-PCI capable hospital (24). However, this is not recommended in patients with NSTE-ACS due to an increase risk in intracranial hemorrhage, fatal, and non-fatal myocardial infarction (25). According to the scientific statement from the American Heart Association/American Stroke Association (AHA/ASA), it is recommended that, in the setting of hyperacute simultaneous cardiocerebral infarction, treatment with intravenous alteplase at the dose appropriate for cerebral ischemia followed by percutaneous coronary angioplasty and stenting is reasonable (26). Although fibrinolytic therapy with intravenous rtPA can be used in both acute ischemic stroke and acute STEMI, different dose requirement and timing of the fibrinolytic therapy after onset hinder the use of rtPA as definitive treatment for both conditions (26). In addition, the higher dose and longer infusion time of rtPA treatment for STEMI when compared with standard dose for acute ischemic stroke may increase the risk of hemorrhagic transformation among patients with simultaneous acute ischemic stroke and myocardial infarction (24, 26). However, the AHA/ASA recommendation did not provide the specific management regarding the acute myocardial infarction subtypes and severity. Despite the longer therapeutic window of coronary reperfusion compared with that of ischemic stroke, an urgent management of coronary reperfusion therapy is required. Prolonged reperfusion therapy can lead to both hemodynamic instability and cardiac complications including hemopericardium, cardiac rupture, and cardiac tamponade. Our goal for treatment of hyperacute simultaneous cardiocerebral infarction is to reperfuse both cerebral and cardiac tissues in a timely manner.

Endovascular therapy is recommended as a combined treatment for acute ischemic stroke in selected patients with large vessel occlusion following the intravenous thrombolytic therapy (27). A meta-analysis from five randomized controlled trials showed that the endovascular treatment significantly improved functional independency compared with intravenous thrombolytic treatment alone with a number needed to treat of 2.6 (28). Since all of the ischemic stroke in the eight reported cases and the two presented cases of hyperacute simultaneous cardiocerebral infarction were due to large vessel occlusions, combination of endovascular therapy and PCI after initial intravenous thrombolytic might be justified for the patients who arrived within the intravenous thrombolytic therapy window for stroke.

**Figure 2:**
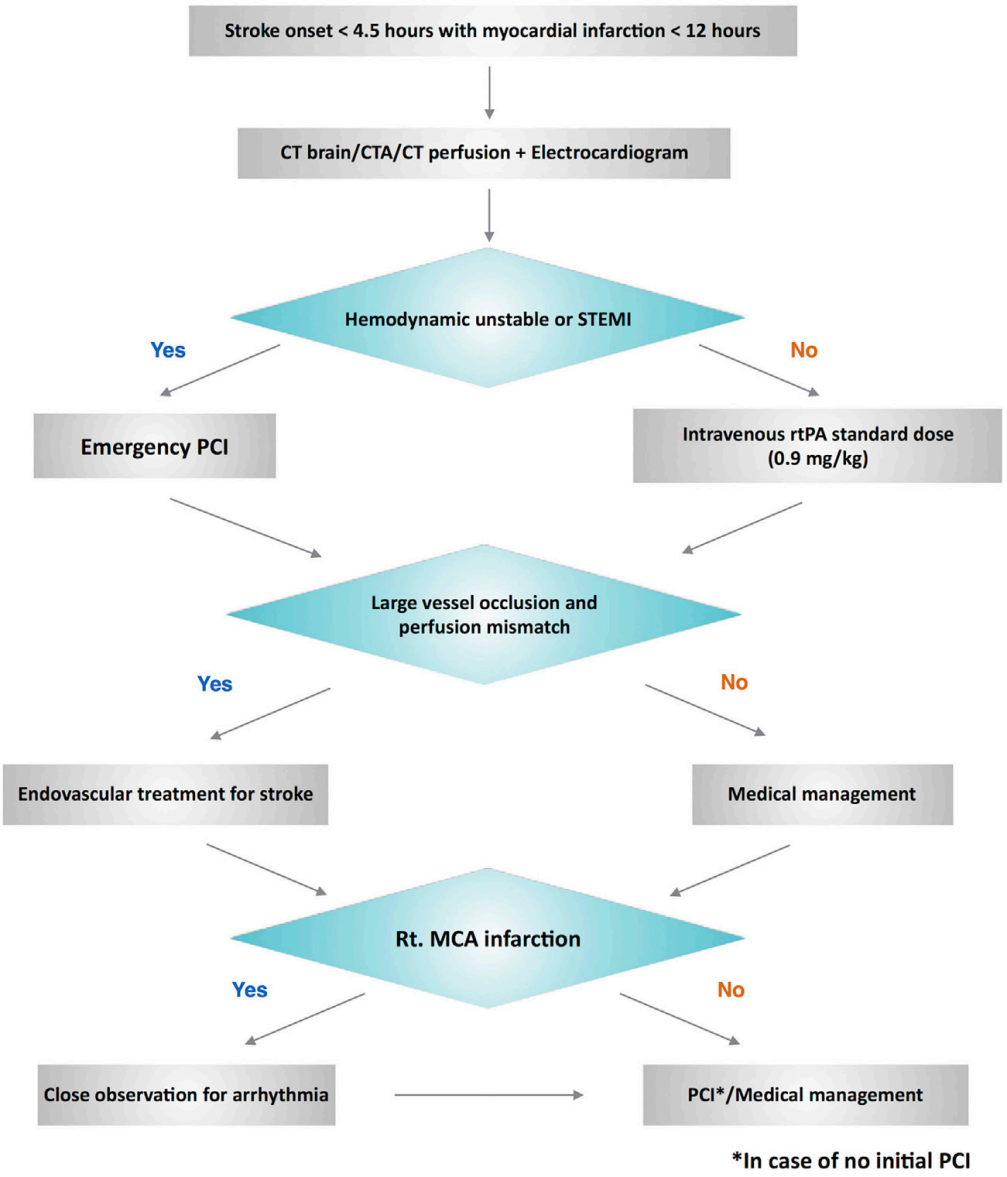
Proposed management algorithm for hyperacute simultaneous cardiocerebral infarction.

We propose a management algorithm (Figure 2) for this challenging scenario of patients with hyperacute simultaneous cardiocerebral infarction who arrive at the hospital within the time window of thrombolytic therapy for acute ischemic stroke. We would like to emphasize the evaluation of the hemodynamic status among these patients, which will influence the decision of whether to treat the cerebral or coronary infarction first. The difference of hemodynamic status of our two patients had an impact on the priority of our management. Case 1 who had stable vital signs was treated with intravenous thrombolytic for acute ischemic stroke followed by PCI for acute myocardial infarction. However, PCI was performed initially in Case 2 due to the hemodynamic instability.

Autonomic dysfunction after acute ischemic stroke is common especially when the insular cortex is involved. The insular cortex is one of the major regulators of the central autonomic network. Lateralization of autonomic dysfunction after acute ischemic stroke has been demonstrated. Lesion in right insular cortex, which is the center of sympathetic control, is associated with parasympathetic overactivity whereas lesion in left insular cortex, the center of parasympathetic control, is associated with sympathetic overactivity (29). These autonomic imbalance can result in myocardial injury, cardiac arrhythmia, and sudden cardiac death (19–21, 29). According to an analysis from the 1,000 Plus database study by Hanne et al., survival rate was significantly lower among patient with insular infarction. Right insular infarction is also significantly associated with mortality (hazard ratio 2.6, CI 1.3–5.4) (30). These results are in accordance with an analysis from the Third International Stroke Trial (IST-3), which demonstrated that mild-to-moderate ischemic stroke defined by NIHSS ≤ 15 in the right insular cortex was significantly associated with death or dependency (31). However, in both studies, there are insufficient data to determine the cause of death among these patients. One of our presented cases with right-middle cerebral artery infarction developed sudden cardiac arrest.

In case of suspected hyperacute simultaneous cardiocerebral infarction, after initial evaluation, non-contrast CT brain, CT angiogram, CT perfusion, and electrocardiogram should be done to confirm the diagnosis. Hemodynamic status is then reevaluated. Emergency PCI is required for the patients who have unstable hemodynamic status or ST-segment elevation myocardial infarction. Subsequent endovascular treatment for ischemic stroke is then performed in case of large vessel occlusion. Those with stable hemodynamics, intravenous rtPA should be administered initially at a standard dose. Endovascular treatment is then performed in case of large vessel occlusion. Since right-middle cerebral artery infarction usually involves the right insular cortex, which is associated with higher mortality (30, 31), we suggest close observation and cardiac monitoring for arrhythmia among these patients with hyperacute simultaneous cardiocerebral infarction. The decision to perform PCI among patients with NSTE-ACS can be based on two treatment strategies: the ischemic-guided strategy and early invasive strategy (25).

## Conclusion

Although uncommon, simultaneous cardiocerebral infarction is among one of the most challenging medical emergency conditions and requires timely management. We propose a management algorithm for the patient based on the hemodynamic stability and suggest close cardiac monitoring based on the site of cerebral infarction in order to facilitate the decision to rescue the brain or the heart first. Future clinical trial study, though difficult to perform due to its rarity, is required in order to develop the optimal management of this catastrophic clinical scenario.

## Ethics Statement

Due to the patient’s condition, written consent was obtained from the patient’s relative for the publication of this manuscript.

## Author Contributions

NK and NS developed the main conceptual idea and contributed to the design of the manuscript. NK wrote the manuscript. AC was involved in manuscript editing. NS supervised, critically reviewed, and edited the manuscript. NK, AC, and NS approved the final version of the manuscript.

## Conflict of Interest Statement

The authors declare that the research was conducted in the absence of any commercial or financial relationships that could be construed as a potential conflict of interest.
